# Electro-Optical Characteristics of Polymer Dispersed Liquid Crystal Doped with MgO Nanoparticles

**DOI:** 10.3390/molecules27217265

**Published:** 2022-10-26

**Authors:** Yuzhen Zhao, Jinqian Li, Yang Yu, Yang Zhao, Zhun Guo, Ruijuan Yao, Jianjing Gao, Yongming Zhang, Dong Wang

**Affiliations:** 1 Xi’an Key Laboratory of Advanced Photo-Electronics Materials and Energy Conversion Device, School of Electronic Information, Xijing University, Xi’an 710123, China; 2 School of Materials Science and Engineering, University of Science and Technology Beijing, Beijing 100083, China

**Keywords:** polymer dispersed liquid crystal, inorganic oxide nanoparticles MgO, electro-optical properties, polymerization induced phase separation

## Abstract

In this paper, inorganic oxide MgO nanoparticles-doped polymer dispersed liquid crystal (PDLC) films were made from a mixture of the prepolymer, SLC1717 liquid crystal, and MgO nanoparticles by the polymerization induced phase separation (PIPS) process. To observe the effect of MgO concentration, PDLC was dispersed with 0.2, 0.4, 0.6, and 0.8 wt.% MgO. Electro-optical properties of the films have been investigated using LCD parameter meter and Scanning Electron Microscope (SEM) at room temperature. It is established that MgO nanoparticles affect the microstructure of PDLC films significantly because of the formed agglomerates of MgO nanoparticles. Results show an improvement in the electro-optical properties and a decrease in the driving voltage for doped systems with MgO nanoparticles. When the doping amount of MgO is 0.8 wt.%, the threshold voltage (V_th_) is reduced to about 7.5 V. Therefore, MgO-doped PDLC is expected to become an excellent choice in the field of energy-saving.

## 1. Introduction

Polymer dispersed liquid crystal (PDLC) is a new type of liquid crystal display device, since the LC molecules can change their orientation under the influence of an applied electrical field [1], they can switch between a highly scattering opaque state and a transparent state [2,3,4]. It has a simple preparation process, low price, and no need for polarizers, and can be used for large-area flexible displays [5,6,7,8]. In addition to the display, PDLC is now also used in electronically controlled smart glass, liquid crystal gratings, optical switches, and other fields [9,10,11,12,13,14,15,16,17]. Its unique electro-optical performance has attracted widespread attention from domestic and foreign researchers.

Advances in the field of nanotechnology have led to nanostructured materials with adjustable optical, electronic, magnetic, and chemical properties [18,19,20,21]. These nanostructured materials are now very suitable for use as additives in PDLC systems to improve the characteristics of the latter [22,23]. In recent years, a large number of reports have witnessed the effectiveness of metal nanoparticles, inorganic oxide nanoparticles, and ferroelectric nanoparticles in changing the physical and electro-optical properties of PDLC [24,25,26,27,28,29]. For example, Martin U et al. [30] studied the impact of functionalized gold nanoparticles on the impedance response of nematic nanoparticle/liquid crystal dispersions in the frequency range of 0.1 Hz~100 kHz. The result shows that nanoparticle doping does not alter the electro-optic response at frequencies above the occurrence of electrode polarization, while it strongly deteriorates the performance in the low-frequency regime. Zhang Y et al. [31] prepared PDLC films doped with ITO nanoparticles by UV-induced polymerization based on a thiol-acrylate system. It is found that the film has a lower driving voltage (20.7 V), a relatively high contrast ratio (8.3), and a better performance with the lowest transmittance. John VN [32] studied flexible BTO-doped polymer dispersed liquid crystal devices with ferroelectric nanoparticles, and found that due to the spontaneous polarization of ferroelectric nanoparticles, the threshold voltage (V_th_) and saturation voltage (V_sat_) of ferroelectric BTO nanoparticles were respectively added. The threshold voltage (V_th_) and saturation voltage (V_sat_) of PDLC were reduced by 85% and 41%.

At present, PDLC film still has problems such as high energy consumption, slow response, low contrast, etc., and there are still certain limitations in practical applications. MgO nanoparticles have the advantages of absorbing ultraviolet light, easy preparation, low price, etc. When they are doped into nematic liquid crystal systems, polymer dispersed liquid crystals with low driving voltage and high contrast can be obtained. In this study, it is found that the viscosity of UV64-5 is relatively large. When 2-EHA was added, the viscosity of the composite decreased. The more 2-EHA was added, the more the viscosity of the system decreased. Therefore, the number of liquid crystal droplets increases and the size decreases. When the LC content is 50%, the polymer mesh size matches the LC concentration in the mesh best. At this time, the CR is maximum, and the PDLC film has good electro-optical properties. Above all, in this work, we present experimental results on electro-optical properties of the SLC1717 nematic liquid crystal doped by MgO NPs. Scanning Electron Microscope and electro-optical measurements have been carried out to examine the effect of MgO NPs on the PDLC films. The results show that the addition of MgO NPs reduces the driving voltage of the PDLC films.

## 2. Materials and Methods

### 2.1. Materials

**Figure 1:**
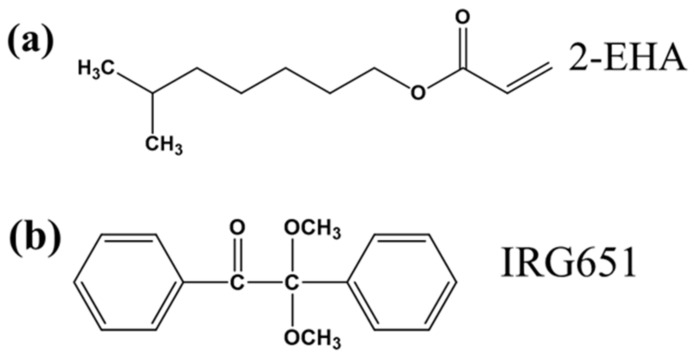
Chemical structures of (**a**) monomer 2-EHA, (**b**) free radical photo-initiator IRG651.

The materials system for fabricating the PDLC films consisted of an ultraviolet (UV) curable polymer UV64-5 (purchased from Shanghai Hanrui Industrial Co., Ltd., Shanghai, China), an acrylic monomer (2-EHA, purchased from Shanghai Macklin Biochemical Technology Co., Ltd., Shanghai, China), free radical photo-initiator (IRG651, purchased from TCI (Shanghai) Development Co., Ltd., Shanghai, China), and nematic LCs (SLC1717, n_o_ = 1.519, n_e_ = 1.720, Δn = 0.201, T_c_ = 92 °C, purchased from Shijiazhuang Chengzhi Yonghua Display Material Co., Ltd., Shijiazhuang, China) with the total mass of 0.5 g. Table 1 lists the typical characteristics of UV64-5. MgO nanoparticles (diameter 40 nm, purchased from Shanghai Naiou Nano Technology Co., Ltd., Shanghai, China) at a ratio of 0.2~0.8% wt.% was added to the materials. Figure 1 shows the chemical structure of these different components.

### 2.2. Sample Preparation

**Figure 2:**
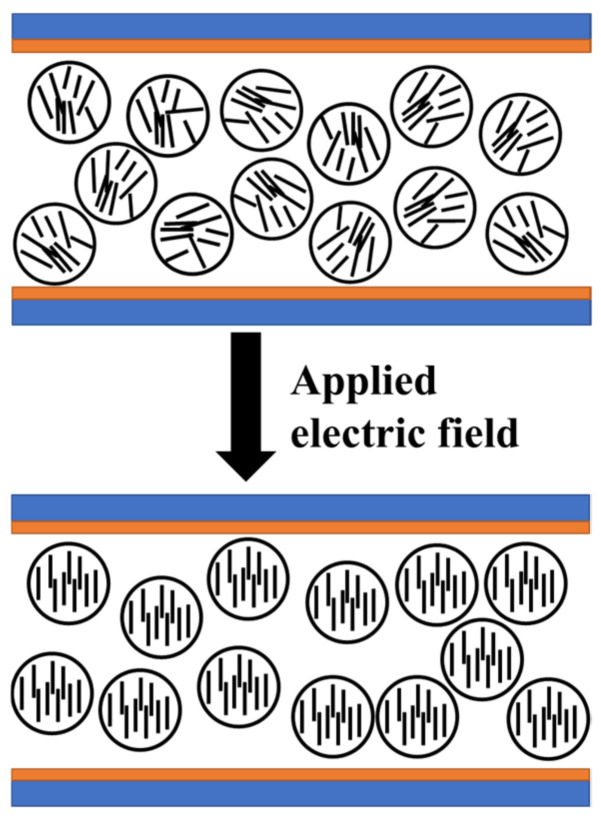
The working principle of PDLC.

Firstly, prepared UV64-5/2-EHA/LC was incubated in an ultrasonic bath for 1 h for 60 °C. After that, MgO at a ratio of 0.2, 0.4, 0.6, and 0.8% wt.% was added to the materials, and the composites were formed. Figure 2 shows the working principle of PDLC.

For homogeneous distribution, UV64-5/2-EHA/LC/MgO composites were mixed using an ultrasonic bath for 3 h at 60 °C. Then composites were injected into ITO coated glass cells with a spacing of 20 μm with the help of capillary action. These prepared samples were exposed to UV light (intensity~5 mW/cm^2^) for 10 min at room temperature to make PDLC films. The compositions of the samples are listed in Table 2.

To study the electro-optical properties of composite materials, the threshold voltage (V_th_), response time (t_off_), contrast ratio (CR), etc., of PDLC were tested by LCD comprehensive parameter meter. The LCD comprehensive parameter meter (LCT-5016) used in this research was produced by Beijing LCD Engineering Research and Development Center. LCD parameter meter is an instrument to test the photoelectric performance of liquid crystal, which can test the photoelectric performance curve (such as threshold voltage), response time, and other photoelectric performance parameters of liquid crystal. To observe the droplet morphology of composites, images of pure and MgO doped PDLC composites were observed by Scanning Electron Microscope (SEM). Before SEM investigated, cyclohexane was prepared to extract LCs from the samples. Samples were immersed in the cyclohexane at room temperature for 7 days. Cyclohexane was evaporated after the samples were opened carefully and lastly, the films were sputtered with gold to observe the structure of the composites under SEM.

## 3. Results and Discussion

### 3.1. UV64-5/2-EHA Ratios Optimization for Non-MgO Doped PDLCs

#### 3.1.1. Morphology of the Films

**Figure 3:**
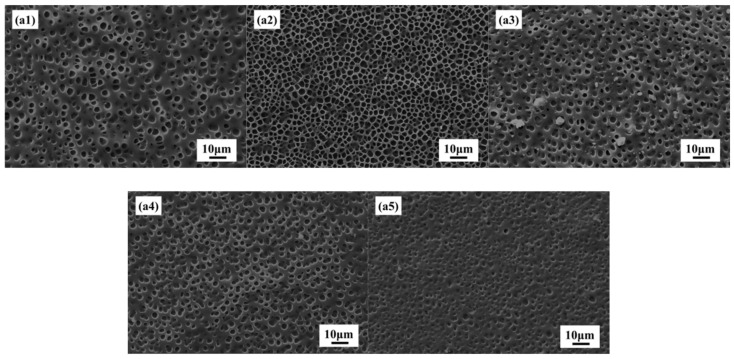
SEM images of (**a1**) 0% 2-EHA; (**a2**) 5% 2-EHA; (**a3**) 10% 2-EHA; (**a4**) 15% 2-EHA; (**a5**) 20% 2-EHA.

The size of the liquid crystal droplets affects the electro-optical properties of the PDLC film material. Therefore, controlling the size of droplets is an important aspect of obtaining good electro-optical properties. Figure 3a1–a5 shows SEM images of PDLC composites with different 2-EHA content 0, 5, 10, 15, and 20%, respectively. In Figure 3, as the 2-EHA content increases, the mesh size in the converged network decreases. The content of UV64-5 is 100, 95, 90, 85, and 80%, respectively. As the content of UV64-5 decreases, the grid size in the converged network decreases. It can be clearly seen from Figure 3 that the reduction of UV64-5 content in PDLC composites will reduce the droplet size. With the decrease of UV64-5 content, the possible reasons for the size reduction can be explained as follows:

It can be seen from Table 1 that the viscosity of UV64-5 is relatively large. After adding 2-EHA, the viscosity of the composite material decreases, and the more the content of 2-EHA added, the more the viscosity of the system decreases. The decrease in the viscosity of the composite material system results in the LC molecules moving more freely during the polymerization process, and the chance of division with each other increases. Therefore, the number of liquid crystal droplets increases and the size decreases. The decrease in LC droplet size with decreased viscosity can also be explained by using the Stokes equation which indicates LC droplet size depending on the viscosity [4,33].

#### 3.1.2. Electro-Optical Performance of PDLC

**Figure 4:**
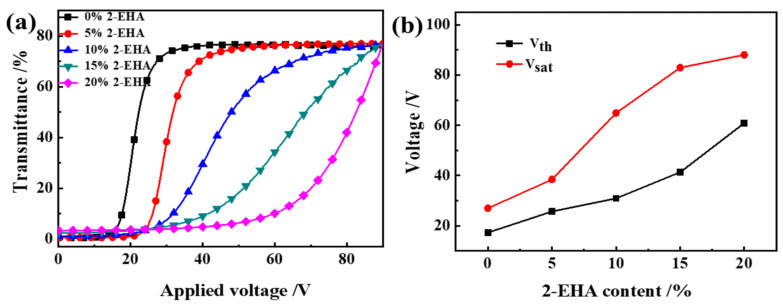

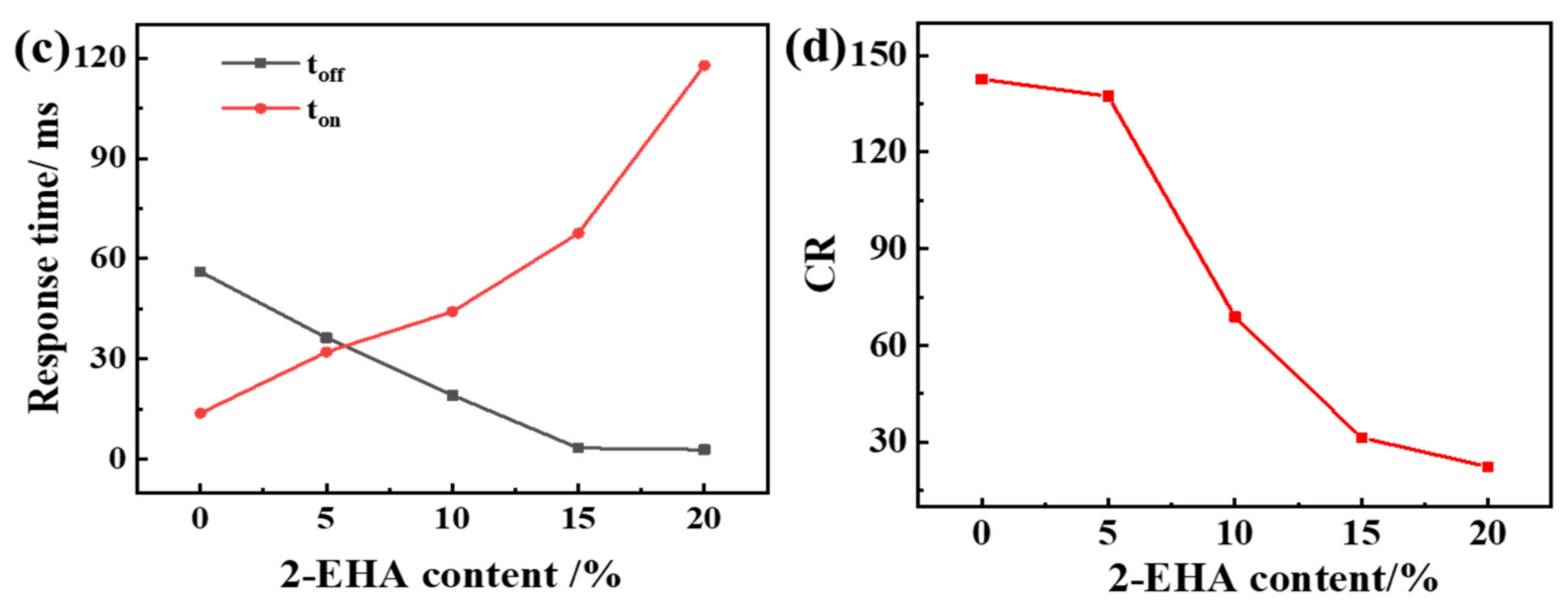
The effect of 2-EHA content on the electro-optical properties of PDLC (**a**) applied electric field (100 Hz) dependence of the transmittance of samples a1~a5; (**b**) V_th_ and V_sat_ of samples a1~a5; (**c**) Response time of samples a1~a5; (**d**) CR of samples a1~a5.

Electro-optical characteristics are an important performance of PDLC, including parameters such as threshold voltage (V_th_), the response time (t_off_), and contrast ratio (CR). V_th_ or V_sat_ indicate that the applied voltage reaches 10% and 90% of the transmittance respectively, and they are generally inversely proportional to the radius of LC droplet (R). The field-dependent transmittance was analyzed using an LCD comprehensive parameter meter at different voltages (0~100 Vac). The voltage-dependent transmittance spectra of the optimized samples, which used air as a reference, is shown in Figure 4a–c.

In Figure 4a, transmittance in the off-state T_off_ increases with the increase of 2-EHA content; transmittance in the on-state T_on_ changes is not obvious. In Figure 4b, V_th_ and V_sat_ increase with the increase of 2-EHA content. In Figure 4c,d, t_off_ and CR decrease with the increase of 2-EHA content and t_on_ increases with the increase of 2-EHA content. The decrease in driving voltage is due to the reduction of the polymer network and the enhanced anchoring effect of the polymer matrix on the liquid crystal droplet molecules, which also makes the liquid crystal molecules oriented in the direction of the electric field when driven by a higher voltage. After the voltage is removed, the interface still has a great effect, the orientation of the liquid crystal molecules becomes easy when the voltage is removed, so the t_off_ is shortened. It can be seen from Figure 4 that when no driving voltage is applied, the off-state transmittance (T_off_) of the sample increases. In this way, the CR defined by T_on_/T_off_ decreases.

### 3.2. LC/Monomer Ratios Optimization for Non-MgO Doped PDLCs

#### 3.2.1. Morphology of the Films

**Figure 5:**
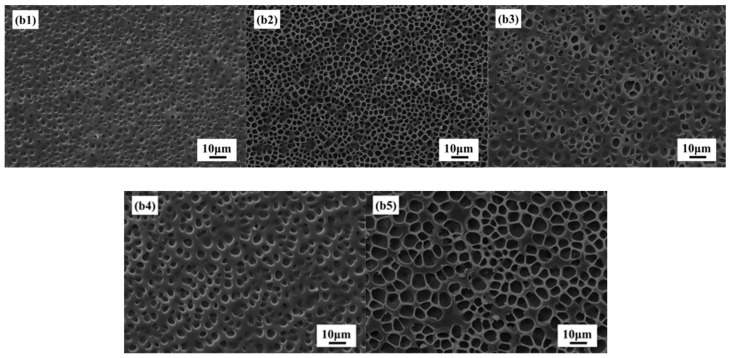
SEM images of (**b1**) 45% LC; (**b2**) 50% LC; (**b3**) 55% LC; (**b4**) 60% LC; (**b5**) 65% LC.

Figure 5b1–b5 shows SEM images of PDLC composites with different LC content 45, 50, 55, 60, and 65%, respectively. It is clear from Figure 5 that increasing the LC content in PDLC composite increases the polymer network. Additionally, with the increase of liquid crystal molecular content, the polymer network mesh will gradually increase. That is because the increase in the LC content directly leads to a decrease in polymerizable monomers in the composite material, which reduces the degree of cross-linking of the polymer network and increases the mesh.

#### 3.2.2. Electro-Optical Performance of PDLC

**Figure 6:**
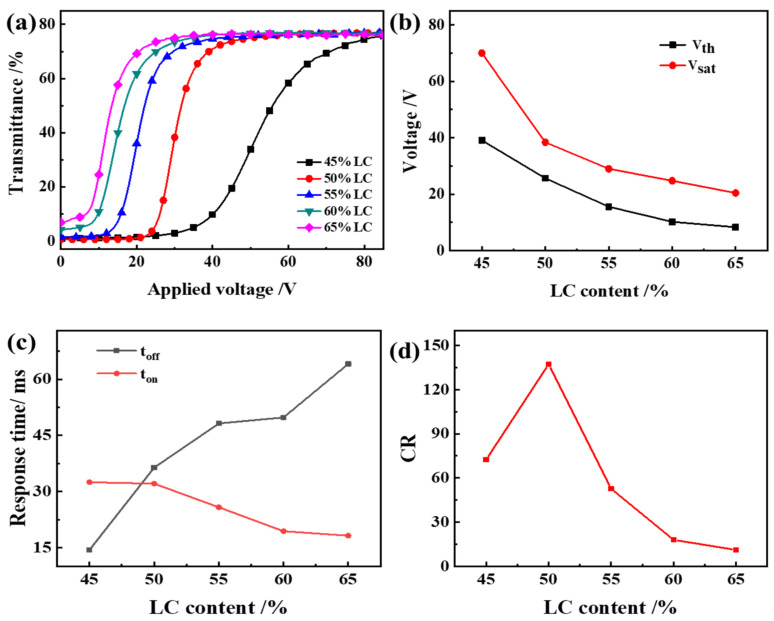
The effect of liquid crystal content on the electro-optical performance of PDLC (**a**) applied electric field (100 Hz) dependence of the transmittance of samples b1~b5; (**b**) V_th_ and V_sat_ of samples b1~b5; (**c**) Response time of samples b1~b5; (**d**) CR of samples b1~b5.

Figure 6 shows the voltage-dependent transmittance spectra of the optimized samples, which used air as a reference. It can be seen from Figure 6 that the V_th_ and V_sat_ of the PDLC films decrease with the increase of LC content, and the t_off_ increases. This is because as the liquid crystal content increases, the proportion of small liquid crystal molecules dissolved in the polymer matrix decreases, and the proportion of small liquid crystal molecules precipitated increases, and the liquid crystal droplets formed by aggregation show an increasing trend. With the increase of the liquid crystal content, the small liquid crystal molecules are more likely to gather into liquid crystal droplets, and the phase separation effect becomes better, so the electro-optical curve of PDLC becomes better. The time required for the liquid crystal droplets to be randomly distributed again is increased, and the t_off_ is increased.

The CR firstly rises and then drops. When LC content is 50%, the polymer mesh size matches the LC concentration in the mesh best, and CR is the largest at this time. When continuing to increase LC content, the CR decreases. This is because with the LC content increases, the mesh size increases, causing the T_off_ to increase, and thus CR decreases. Therefore, when the LC content is 50%, the PDLC film has good electro-optical properties.

From the previous investigation, we can know that when the material ratio is LC/UV64-5/2-EHA = 50%/95%/5%, the PDLC film has excellent performance. Therefore, this was used as a pure sample of MgO doped samples.

### 3.3. The Influence of MgO Content on PDLC Films

#### 3.3.1. Morphology of the Films

**Figure 7:**
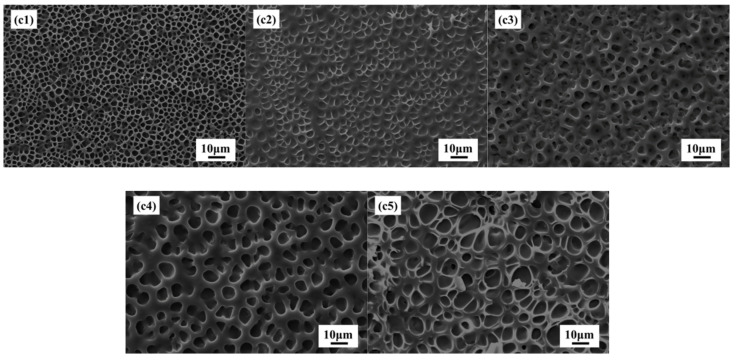
SEM images for (**c1**) pure PDLC; (**c2**) PDLC with 0.2% MgO; (**c3**) PDLC with 0.4% MgO; (**c4**) PDLC with 0.6% MgO; (**c5**) PDLC with 0.8% MgO.

Figure 7c1–c5 shows SEM images of PDLC composites with different MgO nanoparticles content of 0, 0.2, 0.4, 0.6, and 0.8%, respectively. It is clear from Figure 7 that increasing with MgO content in PDLC composite increases the polymer network. The reason for the above phenomenon may be that in the composite containing three components (LC, polymer, MgO NPs), the uniform distribution of all the components is not feasible and it is expected that NPs may have different distributions in the PDLC composite, and most NPs and their aggregates are randomly distributed inside the polymer matrix. However, some NPs are trapped by the defect and are located at the polymer–LC interface. It is expected that the mutual interactions at the polymer–LC interface as well as bulk droplets are changed and it may affect droplet orientation as well as transmission. Compared with pure PDLC materials, the droplet size of PDLC materials doped with nanoparticles is relatively larger, which is mainly because MgO nanoparticles may absorb some UV light required for polymerization. In PDLC composites doped with nanoparticles, more time is needed to complete polymerization. As liquid crystal droplets are allowed more time to condense, form, and grow, the size of the droplets will also increase. On the other hand, the larger droplet size in PDLC doped with nanoparticles may be due to the obstacles caused in the UV polymerization process, where the phase-separated solid polymer and liquid crystal droplets are formed through heterogeneous nucleation and growth processes.

It can be expected that the interaction at the polymer–LC interface and the interaction of the droplets will change, which may affect the orientation and transmittance of the droplets. It is generally believed that the nanoparticles are normally involved in the polymer phase [34], but complete exclusion of nanoparticles from the bulk LC droplets cannot be ruled out. NPs may be present in very small quantities in LC droplets but were not visible in SEM in this study. The presence of NPs changes the polymer matrix and reduces the adhesion between the LC droplets and the polymer matrix [35], resulting in the phase separation process more adequately. Therefore, the mesh size increases with the increase of MgO content.

#### 3.3.2. Electro-Optical Performance of PDLC

**Figure 8:**
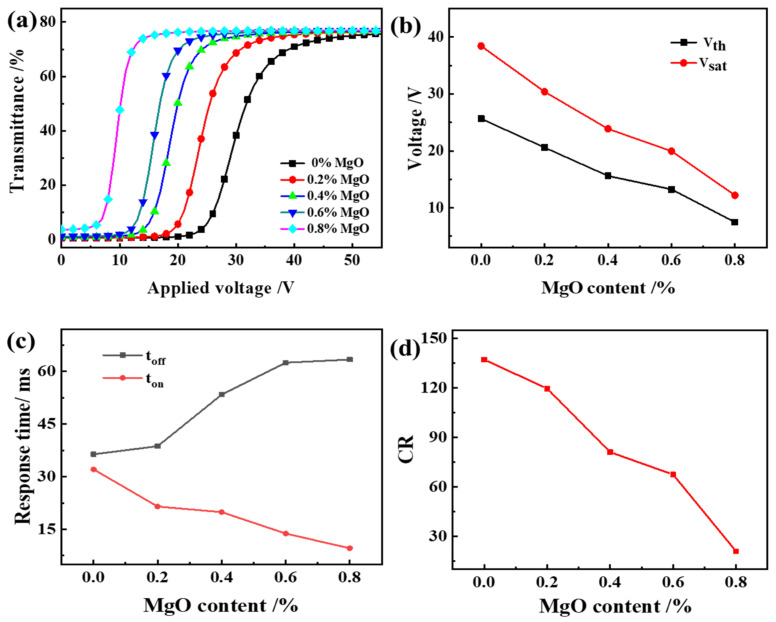
The effect of MgO content on the electro-optical properties of PDLC (**a**) applied electric field (100 Hz) dependence of the transmittance of samples c1~c5; (**b**) V_th_ and V_sat_ of samples c1~c5; (**c**) Response time of samples c1~c5; (**d**) CR of samples c1~c5.

As can be seen from Figure 8, V_th_ and V_sat_ of PDLC films decrease with the increase of MgO content and t_off_ increases. The result is the same as the literature [36]. From the above, we can see that the addition of NPs reduces the adhesion between the LC droplets and the polymer, thereby changing the anchoring energy between them and reducing the driving voltage. On the other hand, as we know, the values of V_th_ = π(K_eff_/ε_0_ ∆ε)^1/2^; ε_0_ is the vacuum permittivity, which is a fixed value; ∆ε is the relative dielectric constant. The reduction of V_th_ may be attributed to the reduction of the order parameters S by doping nanoparticles. From the well-known proportionalities K_eff_ ∝S^2^, ∆ε∝S, then we have V_th_∝$\sqrt{S}$. Thus, the V_th_ may decrease as the order parameter S decreases due to the existence of appropriate nanoparticles [37].

In addition, since the addition of MgO reduces the anchoring force of the polymer matrix to the liquid crystal molecules, after the voltage is removed, the time for the liquid crystal molecules to return to a randomly distributed state increase. The reason for the decrease in CR with the increase of MgO content is the increase in the T_off_ of PDLC as the polymer mesh increases. Therefore, the CR defined by T_on_/T_off_ decreases.

#### 3.3.3. UV Spectra and Photographs of MgO Doped PDLC Film

**Figure 9:**
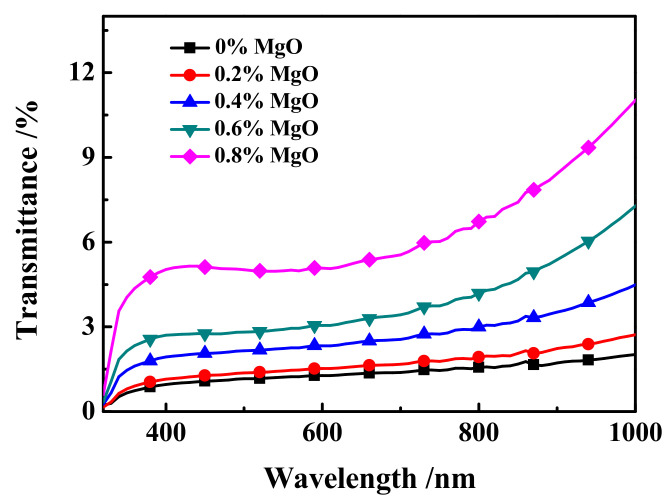
The off-state transmittance (T_off_)-wavelength (λ) curve of samples c1~c5.

**Figure 10:**
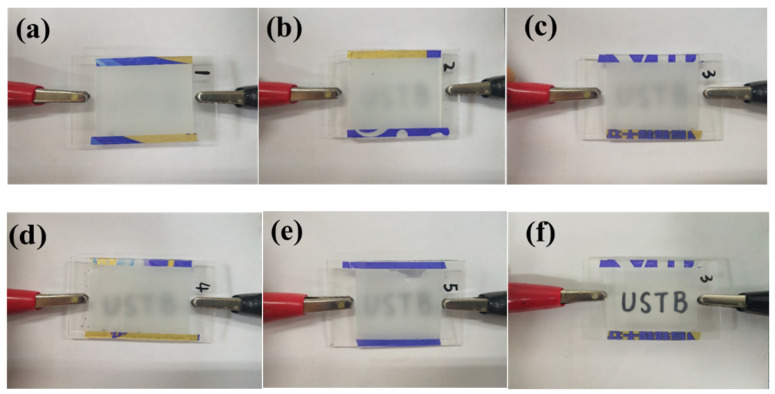
Photographs of samples c1~c5 in the off-state: (**a**) sample c1; (**b**) sample c2; (**c**) sample c3; (**d**) sample c4; (**e**) sample c5; (**f**) sample c3 in on-state photographs.

The off-state transmittance (T_off_)-wavelength (λ) curve of samples c1~c5 at 320~1000 nm is shown in Figure 9. As shown in Figure 9, the transmittance of all samples in the 320 nm~780 nm UV-Visible wavelength range is between 0.15% and 6.5%. In the near-Infrared band 780 nm~1000 nm, the transmittance of the sample is between 1.5% and 11.3%, and the larger the wavelength, the upward trend of T_off_. Moreover, at the same wavelength, the changing trend of the T_off_ of samples c1~c5 is consistent with the actual results of the samples in Figure 10, which is consistent with the electro-optical performance results of the samples shown in Figure 8a.

## 4. Conclusions

MgO nanoparticles were introduced into the PDLC material of UV64-5/2-EHA/SLC1717 formula to change its performance, and were observed through analysis and discussion. The results obtained from electro-optics show that the doped MgO NP system has higher performance, especially in terms of reducing the driving voltage (for example, after doping 0.8% MgO nanoparticles in the composite system, the Vth drops from 25.6 V to 7.5 V). This phenomenon can be explained by morphological SEM results, which show that the size of the LC droplets is different compared to the undoped system. When MgO nanoparticles are added to the composite material, the interaction force between MgO and LC molecules partially replaces the interaction force between the polymer matrix and the LC molecules, and the former is smaller than the latter, resulting in more complete phase separation. Therefore, adding MgO nanoparticles to the composite material will affect the anchoring force at the interface between the LC molecules and the polymer matrix, and reduce the voltage required to reorient the LC molecules.

## Figures and Tables

**Table 1:** Typical characteristics of UV64-5.

Ingredient	Viscosity (cp)	n
methacrylate	340~650	1.505~1.528

**Table 2:** The compositions of the samples.

Sample	LC (wt.%)	UV64-5 (wt.%)	2-EHA (wt.%)	MgO (wt.%)
Group A				
a1	50	100	0	0
a2	50	95	5	0
a3	50	90	10	0
a4	50	85	15	0
a5	50	80	20	0
Group B				
b1	45	95	5	0
b2	50	95	5	0
b3	55	95	5	0
b4	60	95	5	0
b5	65	95	5	0
Group C				
c1	50	95	5	0
c2	50	95	5	0.2
c3	50	95	5	0.4
c4	50	95	5	0.6
c5	50	95	5	0.8

## Data Availability

Not applicable.
